# Unique algorithm for the evaluation of embryo photon emission and viability

**DOI:** 10.1038/s41598-024-61100-8

**Published:** 2024-07-02

**Authors:** József Berke, István Gulyás, Zoltán Bognár, Dávid Berke, Attila Enyedi, Veronika Kozma-Bognár, Péter Mauchart, Bernadett Nagy, Ákos Várnagy, Kálmán Kovács, József Bódis

**Affiliations:** 1 National Laboratory on Human Reproduction, University of Pécs, Pécs, Hungary; 2 Department of Drone Technology and Image Processing, Dennis Gabor University, Budapest, Hungary; 3 Department of Medical Biology and Central Electron Microscope Laboratory, Medical School, University of Pecs, Pécs, Hungary; 4 HUN-REN–PTE Human Reproduction Research Group, Pécs, Hungary; 5 John Von Neumann Computer Society, Multimedia in Education Section, Budapest, Hungary; 6 Dennis Gabor University, Rector’s Cabinet, Budapest, Hungary; 7 Department of Obstetrics and Gynecology, Medical School, University of Pecs, Pécs, Hungary

**Keywords:** Photon emission, Entropy-weighted spectral fractal dimension, Spectral fingerprint, Mouse embryo, Fractal structure, Entropy level energy, Biophysics, Medical research, Optics and photonics, Physics

## Abstract

Living cells have spontaneous ultraweak photon emission derived from metabolic reactions associated with physiological conditions. The ORCA-Quest CMOS camera (Hamamatsu Photonics, Japan) is a highly sensitive and essential tool for photon detection; its use with a microscope incubator (Olympus) enables the detection of photons emitted by embryos with the exclusion of harmful visible light. With the application of the second law of thermodynamics, the low-entropy energy absorbed and used by embryos can be distinguished from the higher-entropy energy released and detectable in their environment. To evaluate higher-entropy energy data from embryos, we developed a unique algorithm for the calculation of the entropy-weighted spectral fractal dimension, which demonstrates the self-similar structure of the energy (photons) released by embryos. Analyses based on this structure enabled the distinction of living and degenerated mouse embryos, and of frozen and fresh embryos and the background. This novel detection of ultra-weak photon emission from mouse embryos can provide the basis for the development of a photon emission embryo control system. The ultraweak photon emission fingerprints of embryos may be used for the selection of viable specimens in an ideal dark environment.

Living cells (e.g. those making up plants, animals and humans) have spontaneous ultraweak photon emission (UPE), the origin of which is linked directly to reactive oxygen species^1,2^. Variations in UPE intensity are associated with physiological and pathological conditions such as thermal, chemical and mechanical stresses, the mitochondrial respiratory chain, the cell cycle and cancerous growth^3–5^.

Worldwide, more than 8 million children have been born as a result of assisted reproductive technology (ART), and about 2.5 million in-vitro fertilisation (IVF) cycles resulting in 500,000 children are delivered annually. The method used for embryo selection and transfer is a very important ethical, and even more so practical, issue. With time-lapse technology and stable culture conditions, embryos can be viewed and monitored continuously throughout their development^6^, but visible light (400–700 nm) is required. The ART success rate remains unsatisfactory, lagging far behind the theoretical possibility, and early findings revealed the damaging effects of visible light on embryos and gametes. This effect, however, remains less well known than the toxic effect of ultraviolet light on living cells.

Early embryonic development is characterised by rapid cell division and embryonic gene activation, which make embryos extremely vulnerable and sensitive to environmental influences. The human body protects itself against visible light by reducing the radiation dose. However, light can damage cells during IVF, especially intracytoplasmic sperm injection (ICSI), as well as during oocyte and sperm preparation, embryo incubation, microscopic examination and embryo transfer^7–9^. To protect against such damage during laboratory procedures, we created a dark environment by covering the devices used for manipulation during IVF/ICSI procedures and applying red filters to laboratory, microscope and IVF workstation light sources. The fertilisation, blastocyst development and clinical pregnancy rates were significantly higher for embryos protected from light than for those manipulated in conventional lighted conditions^7^. Thus, the creation of a dark environment and use of light filters can reduce harmful environmental effects in IVF laboratories^7–10^.

The ever-increasing role of IVF in human reproduction and the need for the establishment of applied methodology make the development of methods based on the latest scientific evidence a moral obligation. Cell photon emission may be employed to eliminate the risks associated with visible light. Due to the extremely low energy of UPE, however, the feasibility of detecting embryo photon emission under the conditions applied during embryo development needs to be considered. For this purpose, we explored the detection of photon emission from mouse embryos and viable embryo selection based thereon.

## Results

**Figure 1:**
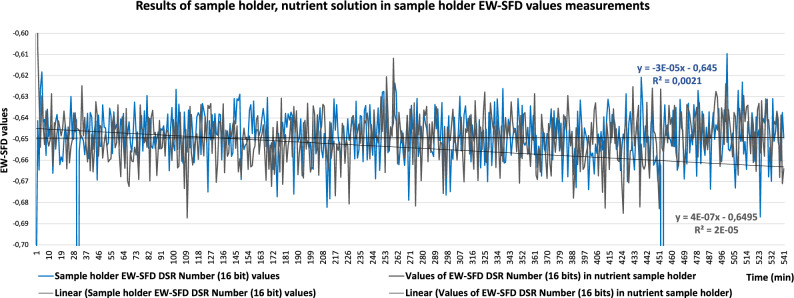
Reference measurements obtained with the sample holder with and without nutrient solution in the measurement space based on entropy-weighted spectral fractal dimension (EW-SFD) values (Methods, Eq. 11). No significant difference was observed between measurements, indicating that the nutrient solution had a minimal effect on the measurement results.

**Figure 2:**
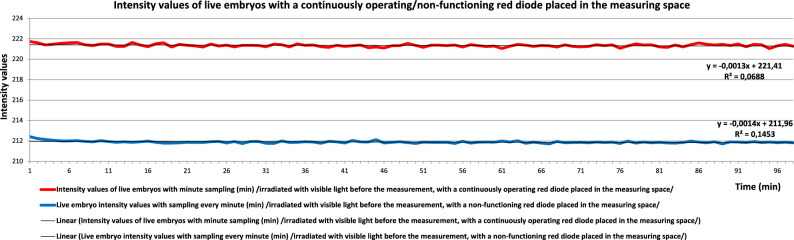
Intensity measurements obtained from live embryos with the non-working (blue) and continuously working (red) diode. Data were corrected using data obtained with the sensor operating at − 20 °C in the dark.

**Figure 3:**
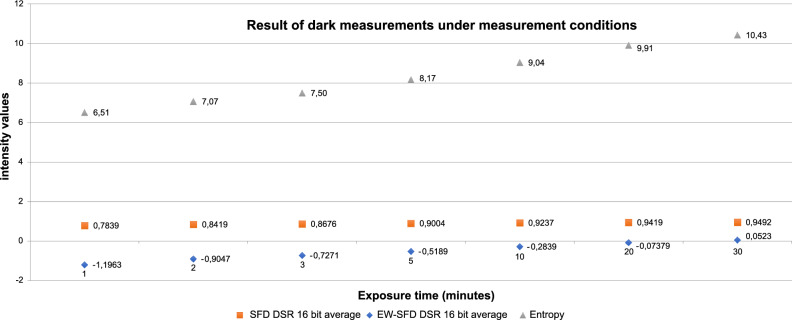
Intensity values for the information content (entropy), spectral fractal structure^11^ and entropy-weighted spectral fractal structure (measurements obtained in the dark) as functions of integration times.

We successfully detected spontaneous photon emission from developing mouse embryos under ideal incubation conditions (i.e. without external stimulation) using a special light-free setup created with a photon camera (Hamamatsu Photonics, Japan) and microscope incubator (Olympus). To evaluate the higher-entropy energy data, we developed a unique algorithm that shows the self-similar structure of the photon energy released by the embryos. No significant difference was observed between reference measurements taken with the sample holder with and without embryo incubation medium in the measurement space, indicating that the medium had a minimal effect on the results (Fig. 1). Data obtained from the embryos when the red diode was in the measurement space but not working and those obtained when it was working continuously differed clearly (Fig. 2), but it was not possible to determine the extent of the influence of noise (effects of the sample holder, nutrient solution and/or embryo). The information content (entropy), spectral fractal structure and entropy-weighted spectral fractal structure, shown in Fig. 3 as functions of the integration time, supported the success of physiologically justified sampling. We used an integration time of 1 min and sampling areas of 21 × 21 pixels.

**Figure 4:**
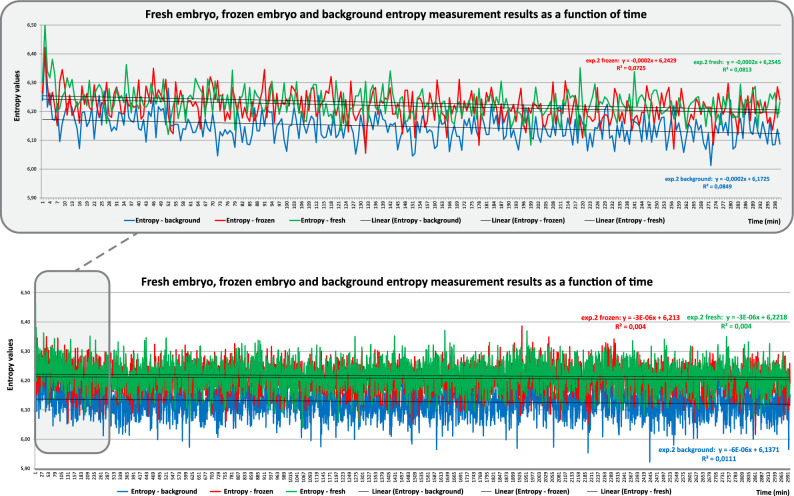
Entropy values for the background and fresh and frozen embryos, obtained over 50/5 h with a 1-min integration time.

**Figure 5:**
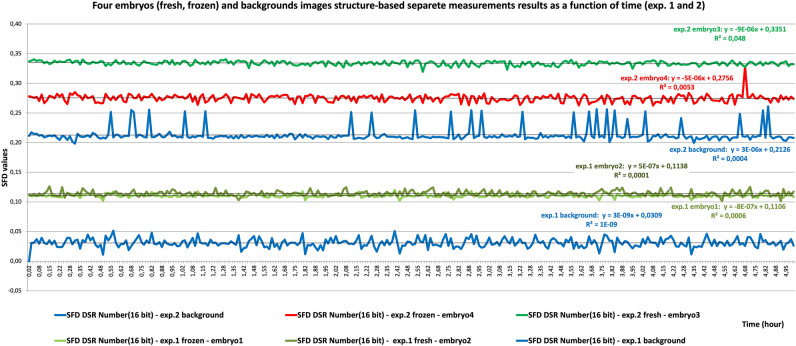
SFD values for the backgrounds, four embryos (fresh and frozen), obtained over 5 h with a 1-min integration time.

Fresh embryo, frozen embryo and background samples could be distinguished clearly by significant differences in entropy values (Fig. 4). Spectral fractal dimension (SFD) values obtained over 5 h of continuous time-lapse recording of live embryos in the eight-cell to blastula stages with 1-min exposure times are provided in Fig. 5. Figure 5 further shows SFD values of two fresh dividing and two frozen dividing embryos where samples were at the 8-cell to 16-cell and morula to blastula stages of development, respectively. The data depth for experiment 1 was 6 bits and the data depth for experiment 2 was 8 bits, which is clearly supported by the SFD values in Fig. 5. The difference regarding the information content of the measurements is well illustrated by the distance between the curves.

**Figure 6:**
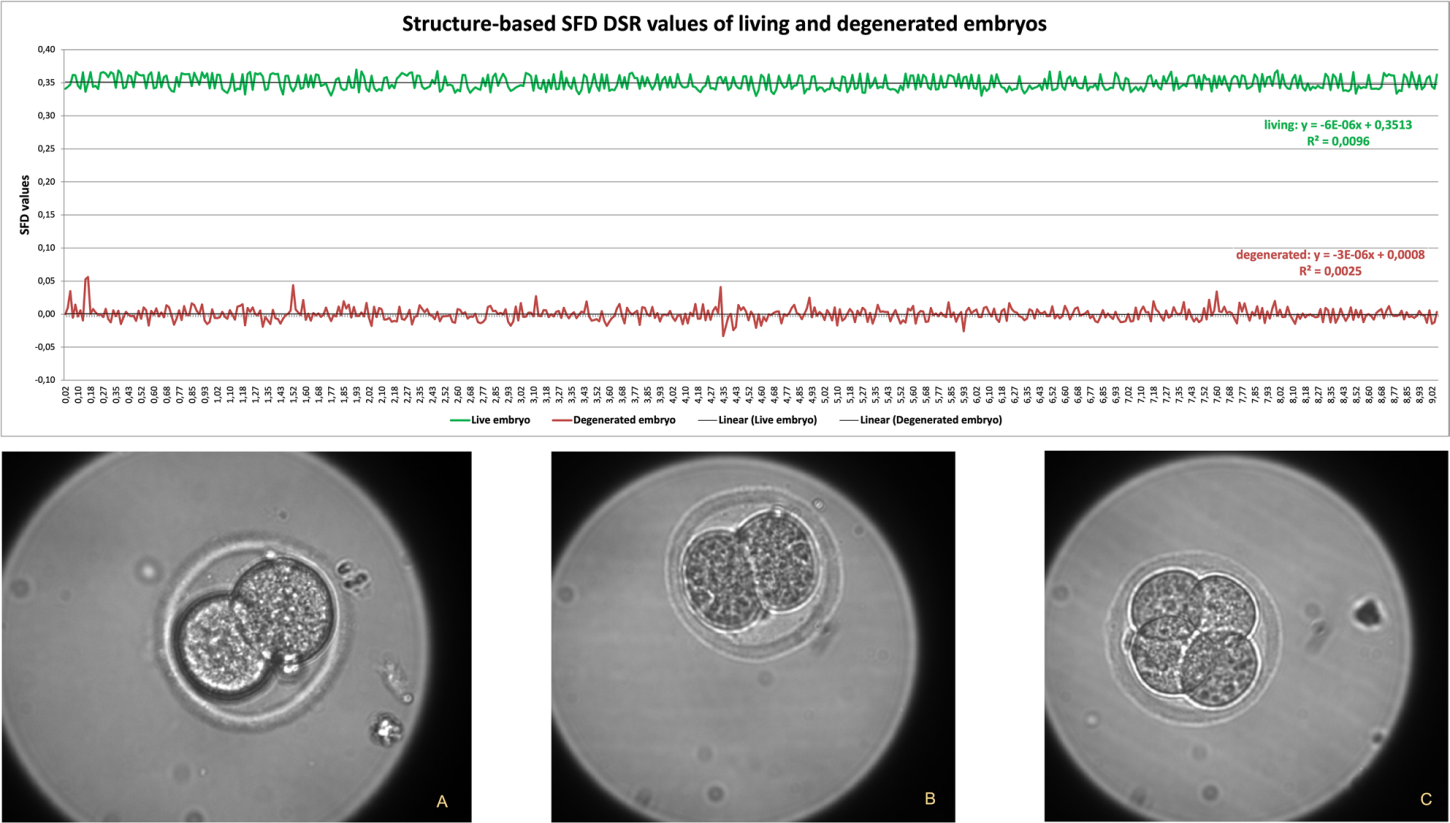
SFD DSR values for living and degenerated embryos, obtained over 9 h with a 1-min integration time (**A**—degenerate at the beginning of the measurement, **B**—degenerate at the end of the measurement, **C**—alive during the measurement).

Living and degenerated embryos could be distinguished clearly by their SFD-DSR (Different Spectral Resolution) structure–based curves calculated based on the Eq. (7) relationship discussed in Methods section (Fig. 6).

## Discussion

In this study, we developed an algorithm for the calculation of entropy-weighted (EW)-SFD values and a setup for the detection of photon emission from developing mouse embryos in the absence of visible light. We confirmed that the presence of the sample holder filled with incubation medium had no significant effect on detection, and showed that freshly conceived embryos emit significantly more photons than do frozen/thawed embryos. Moreover, we showed that living and degenerated embryos could be distinguished via analysis based on the self-similar image structure.

Methods based on light, laser, chemical and other forms of stimulation have been employed in previous photon emission studies^12–21^. Such stimulation cannot be used with embryos, which require maximal protection against all physical, chemical and biological factors.

Equation (8) is based on the relationship described by Eq. (5), and these equations can be used when the useful signal occurring in a random sample is self-similar or has a non-random structure. The algorithm developed here is for the measurement of lossless digital images. It can be used to measure multi-band images per band or together in the case of the representation of 1–16 bits/band, not only with standard pixel representation (8 or 16 bits/pixel), but also with the representation of the maximum number of real pixels in the image.

Equation (5) has been applied in practice in many cases. As it is a metric, it can be used in clustering and supervised classification tasks during metric classification^22^. Berke and colleagues^23,24^ used it to distinguish infected and healthy parts of potato tubers, to classify chip samples and to perform classification tasks on multi- and hyperspectral space images, highlighting its applicability to aerial and space images with more than 20 independent spectral bands. In addition, Berke^22^ supplemented the equation, proposing a spatial and spectral resolution range factor applicable to digital images produced by CCD/CMOS sensors with finite resolution, which includes all three important sensor characteristics (number of pixels, spectral resolution and number of channels). Equation (5) has also been used successfully to detect the effect of pollution on corn via the examination of proximity-derived pollutants (cadmium and carbon black)^25^. Berke^26,27^ applied the equation to recognise potato varieties that cannot be distinguished by eye and to measure leaf damage. Karydas^25^ used Eq. (5) in the mathematical presentation of natural processes containing scale changes.

Biologists have long known about the luminescence of living organisms. A new field of research focuses on biophotons, emitted by living cells in quantities too small to be seen. In research conducted in a dark laboratory, Mayburov^21^ found that biophotons emitted from older loach eggs inhibited the growth of immature eggs. Such radiation had previously been found to communicate between distant samples, resulting in the synchronisation of their development. Mayburov^21^ recognised a binary pattern in the biophoton data, with periodic bursts that provided a clue about such communication; the pattern resembled human transmissions over noisy communication channels, which may be similar to the exchange of binary-coded data through channels in computer networks.

Popp et al.^18^ assumed that biophotons (i.e. UPE) presented a wide variety of frequencies that originate from DNA. He also reported that biophotons are coherent and suggested that they regulate organisms’ life processes. However, the idea that UPE is coherent is under debate. Whether UPE is a byproduct of biological metabolism or has some informational or functional role, and the spectral fingerprints of embryos, remain unclear. More research is needed to determine, for example, how cells produce photons, whether other cells sense them and, if so, how they react.

## Conclusion

Here, we detected UPE from mouse embryos, providing the basis for an embryo monitoring system with the control of developmental, physiological and energetic processes under ideal dark incubation conditions with no external physical or chemical stimulation. We developed a unique algorithm for EW-SFD calculation that demonstrates the self-similar structure of the energy released by embryos in the form of photons. Analyses based on the self-similar image structure enabled the distinction of living and degenerated embryos, as well as frozen and fresh embryos and the background. We hope that these results will serve as a basis for the development of a photon emission embryo control system, given the known harmful effect of visible light on embryos. Embryos’ UPE fingerprints may be used for the selection of viable specimens under ideal dark incubation conditions, but further research and innovation are needed to reach this goal.

## Methods

### Theoretical considerations

The physical basis of our work is the second law of thermodynamics, which states that the total entropy of a closed system does not decrease (Clausius’ formulation):

1 $${\text{dS}} \ge 0.$$

where d*S* is the (physically known) entropy change.

Another fundamental characteristic of biological systems is that they consume energy. From a thermodynamic perspective, however, this energy must have low entropy, i.e. be far from thermal equilibrium. Under ideal conditions (i.e. the absence of external environmental influences), an embryo on incubation medium can be considered to be a thermodynamically closed system. We assumed that the entropy of the energy absorbed by the embryo from the nutrient solution can be divided into low [E_ELE_ (t)] and high [E_EHE_ (t)] parts:

2 $${\text{E}}_{{\text{E}}} \left( {\text{t}} \right) = {\text{E}}_{{{\text{ELE}}}} \left( {\text{t}} \right) + {\text{E}}_{{{\text{EHE}}}} \left( {\text{t}} \right).$$

Embryos’ cells incorporate and use low-entropy energy, which cannot be detected directly in the measurement space we created. Their high-entropy energy can be detected and, based on our assumptions is given off mostly in electromagnetic form^28,29^.

### Algorithm development

We chose parameters for data evaluation that corresponded partly to the embryos’ high-entropy energy output and partly to the sensors’ image data. A digital characteristic suitable for the measurement of entropy-based quantities we selected based on expectations regarding living organisms’ energetic processes and the imaging equipment’s digital data recording. We measured information theory entropy, which has been practically applied to image data in many cases^22–24,26,30,31^.

Rényi^17,32^ defined entropy mathematically in 1961, and a programme for its measurement in 16-bit image data has been developed^26^. Shannon^33,34^ introduced the currently used information-theory concept of entropy in 1948 and then illustrated it with a practical example. According to these definitions, the average information content (entropy) of independent digital messages can be determined by:

3 $$H = \mathop \sum \limits_{i = 1}^{m} p_{i} ld\left( {\frac{1}{{p_{i} }}} \right),$$

where *H* is the information-theoretic entropy and *p*_*i*_ is the probability of occurrence of the *i*th message (relative frequency in practice). The information-theoretic entropy of a closed system can take the following values:

4 $$0 \le {\text{H}} \le {\text{log}}_{2} {\text{n}},$$

where *n* is the number of possible messages. Entropy is smallest when the source always sends the same message, i.e. the image has a single intensity value. It is greatest when the probability of all messages is equal.

When the self-similar (fractal) data structure is of interest in addition to the information content (entropy), the measurement of independent structural parameters is advisable. As our assumption holds that the embryo should be treated as such an object, and according to our previous research on the operation of living objects^24,26,27^, the structural parameter of the fractal dimension was measured. In this case, the values of pixels reaching the sensor from the embryo also contain three essential elements: the intensity of the photon emitted by the embryo, the structure of the photons emitted at different moments during the integration time and noise. We developed a unique EW-SFD function (algorithm) to separate these data from a single region and background. As this function is a measure of the self-similar structure weighted by the entropy of the image data, it encompasses the structure and information content of the energy emitted by the object.

The dimension of a fractal curve is a number that shows how the distance between two selected points on the curve increases when the resolution is increased. Thus, if the topological dimensions of the line and the surface are always 1 and 2, respectively, the fractal dimension falls between these values. Curves and surfaces in the real world are not real fractals; they were created by processes that can create shapes only within specific size ranges. Thus, the dimension can change depending on the resolution. Change can aid the understanding of the processes characteristic of living biological systems involved in creation.

**Figure 7:**
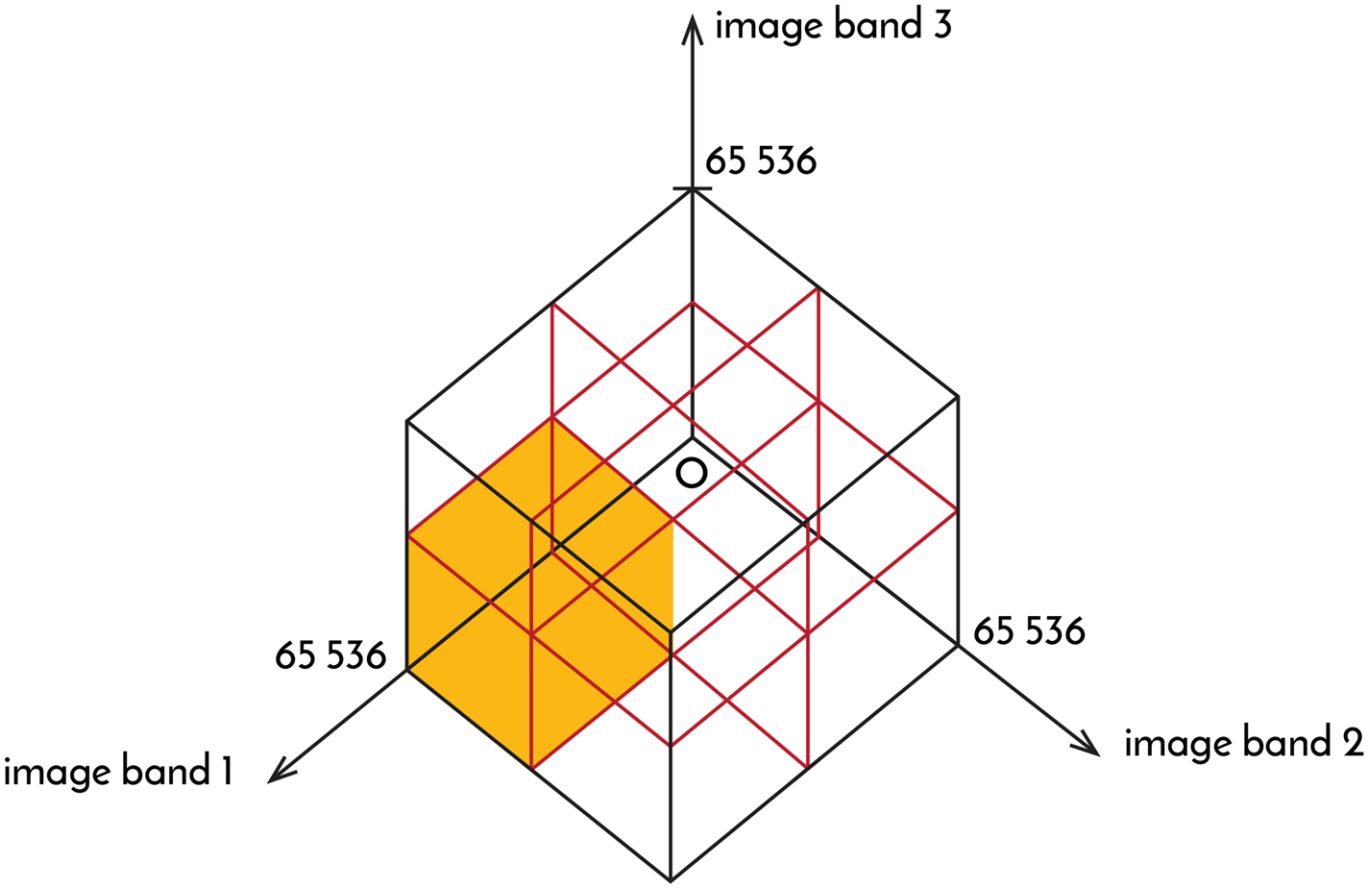
Illustration of spectral boxes (216 = 65,536) used during SFD calculation of 16-bit images. The coordinate system formed by the independent axes (image band 1, 2, 3) forms the spectral space. Each pixel is assigned to the spectral boxes based on the values of the pixels occurring in the same position in the image.

Several methods that are suitable for the calculation of fractal dimensions have been developed^11,35–37^. Such is also the case for SFD calculation^26^, a structural analytical procedure derived from the measurement of general fractal dimensions that represents a novel application of fractals. In addition to spatial structure measurement, SFD calculation is suitable for the measurement of spectral band colour structures and provides sufficient information about the fractal properties of colours and shades. For the calculation of SFD values for two or more image bands with the same spectral resolution (Fig. 7), the following formula involving the simple mathematical averaging of the measured data as a function (number of valuable spectral boxes among all spectral boxes) can be applied:

5 $$SFD_{ESR} = \frac{{n \times \sum\limits_{j = 1}^{S - 1} {\frac{{\log \left( {BM_{j} } \right)}}{{\log \left( {\left( {2^{S} } \right)^{n} } \right)}}} }}{S - 1}$$

where *n* is the number of image layers or channels, *S* is the spectral resolution in bits and *BM*_*j*_ is the number of spectral boxes containing a valuable pixel for bit *j*. The number of possible spectral boxes for bit *j* (*BT*_*j*_) can be calculated as follows:

6 $$BT_{j} = \left( {2^{S} } \right)^{n} .$$

In the case of a single image band, the intensity values of the pixels are considered spectral resolution. This SFD_ESR_ (Equal Spectral Resolution) metric is non-negative definite and symmetrical, and it satisfies the triangle inequality^26^. An additional condition for the performance of the measure is the fulfilment of the regularity condition (i.e. that points on a discrete image plane have a uniform density). In practice, before the analog/digital converter, the image function is subjected to non-linear transformation, making the density of the image function constant. Thus, the condition of regularity usually is or can be considered to be fulfilled for digital images. As the SFD_ESR_ correlation is a metric, it can be used to measure image data exactly.

To calculate the EW-SFD, we begin with the measurable definition of the SFD^26^:

7 $$SFD_{measurement} = \frac{{n\sum\limits_{j = 1}^{S - 1} {\frac{{\log \left( {SBM_{j} } \right)}}{{\log \left( {SBT_{j} } \right)}}} }}{S - 1},$$

where *SBM*_*j*_ is number of spectral boxes containing a valuable pixel for bit *j* and *SBT*_*j*_ is the total possible number of spectral boxes for bit *j*. We weight the number of spectral boxes using an entropy-based weighting factor (*f*_*j*_):

8 $$EW - SFD_{measurement} = \frac{{n\sum\limits_{j = 1}^{S - 1} {\frac{{\log \left[ {f_{j} \times \left( {SBM_{j} } \right)} \right]}}{{\log \left( {SBT_{j} } \right)}}} }}{S - 1}.$$

The possible number of spectral boxes for bit* j* can be calculated according to Eq. (7). The entropy-based weighting factor is calculated as:

9 $$f_{j} = \frac{{H\left( {SBM_{j} } \right)}}{{\max H_{j} }} = \frac{{H\left( {SBM_{j} } \right)}}{j} = \frac{{\sum\limits_{k = 1}^{{2^{S} }} {p_{k} ld\left( {\frac{1}{{}}p_{k} } \right)} }}{j},$$

where max *H*_*j*_ is the maximum entropy for *j* bit pixels, *j* represents the values of the pixels in the *SBM*_*j*_ box in bits, $p_{k}$ is the relative frequency of the *k*th pixel in the *SBM*_*j*_ box and $H(SBM_{j} )$ can be calculated for independent pixels as:

10 $$H\left( {SBM_{j} } \right) = \sum\limits_{k = 1}^{{2^{S} }} {p_{k} ld\left( {\frac{1}{{p_{k} }}} \right)} .$$

Finally, the EW-SFD is calculated as:

11 $$EW - SFD_{measurement} = \frac{{n\sum\limits_{j = 1}^{S - 1} {\frac{{log\left[ {\frac{{\left[ {\sum\limits_{k = 1}^{{2^{S} }} {p_{k} ld\left( {\frac{1}{{p_{k} }}} \right)} } \right] \times (SBM_{j} )}}{j}} \right]}}{{log(SBT_{j} )}}} }}{S - 1}.$$

### Animals

Thirty 7-week-old female and twenty 9-week-old male CD1 mice were purchased from Charles River (Germany). The animals were housed in a Uniprotect Ng/M animal-keeping cabinet (Zoonlab Gmbh, Germany) at 24 °C with a 12/12-h day/night cycle and 50% humidity before the experiments. They were given a minimum of 2 weeks’ accommodation time.

### Superovulation, embryo retrieval and culture

Eight- to 12-week-old female CD1 mice were injected with 5 IU follicle-stimulating hormone (Merional, IBSA Pharma, Switzerland). Forty-eight hours later, the mice were treated with 5 IU luteinizing hormone (Chloragon, Ferring, Hungary) and placed directly in CD1 males. Two days later (1.5 days post-coitum), embryos in the two- and four-cell stages were flushed from the fallopian tubes and cultured in groups (10–14/50-μl droplet) in potassium simplex optimisation medium (Millipore, England) supplemented with 0.4% bovine serum albumin under mineral oil at 37 °C with 5% CO_2_ in the air. The culture media were replaced after 2 days. UPE measurements were performed on two-cell embryos and after 24 h culture at the four–eight-cell stage. Only high-quality fresh viable embryos were used as fresh embryos.

After the 24-h cleavage, the two-cell stage non-dividing embryo was considered dead and was used as a degenerated embryo for the measurements.

#### Method of obtaining frozen embryos

Good-quality 6‒8-cell-stage embryos were vitrificated and warmed for the measurements. The process of vitrification and warming was performed by the Rapid-i vitrification and Rapid-i warming sets (Vitrolife AG Gothenburg, Sweden). The rate of the live embryos after warming was ˃ 90%. The embryos were measured after one or two hours of a post-warming culture (37 °C). Some of the eight-cell stage embryos was used for the investigation.

**Figure 8:**
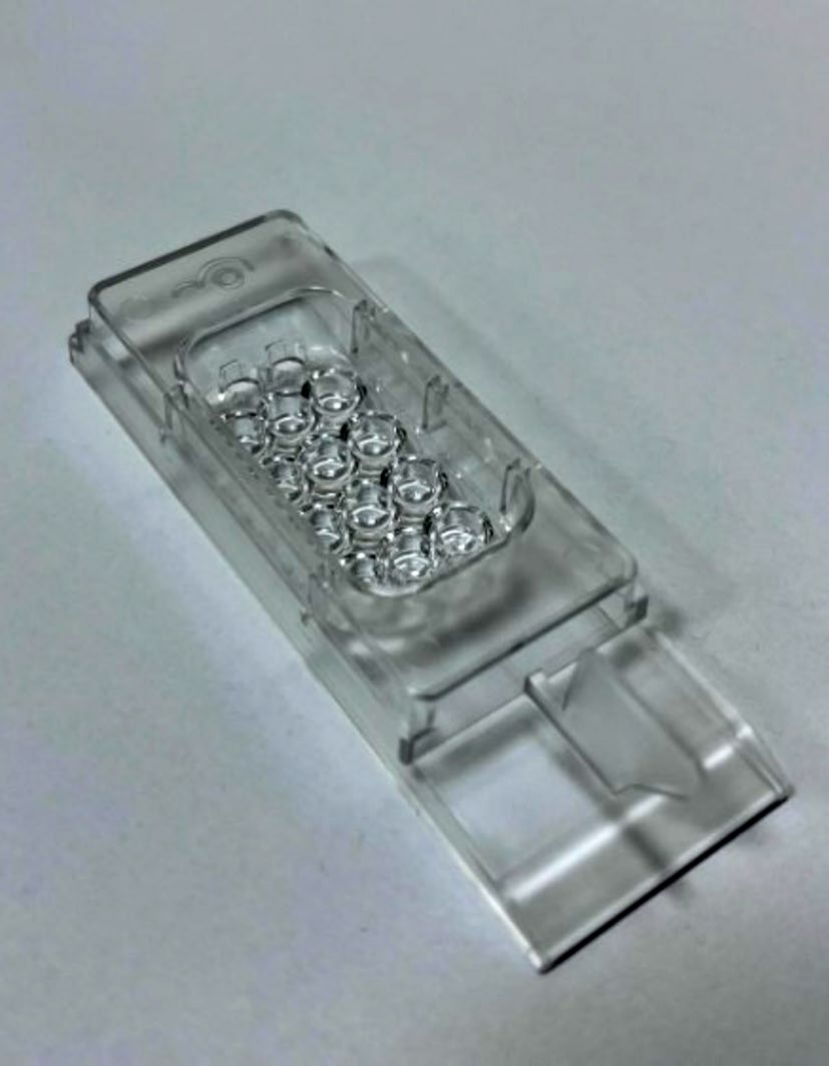
Embryo culture dish.

UPE detection was performed after the embryos had been cultured for 1 or 2 h in EmbrioSlide dishes (Vitrolife; Fig. 8), prepared according to the manufacturer’s recommendation. The dishes’ microwell design permitted the embryos to be halted in their original positions during data collection.

### Photon emission detection

**Figure 9:**
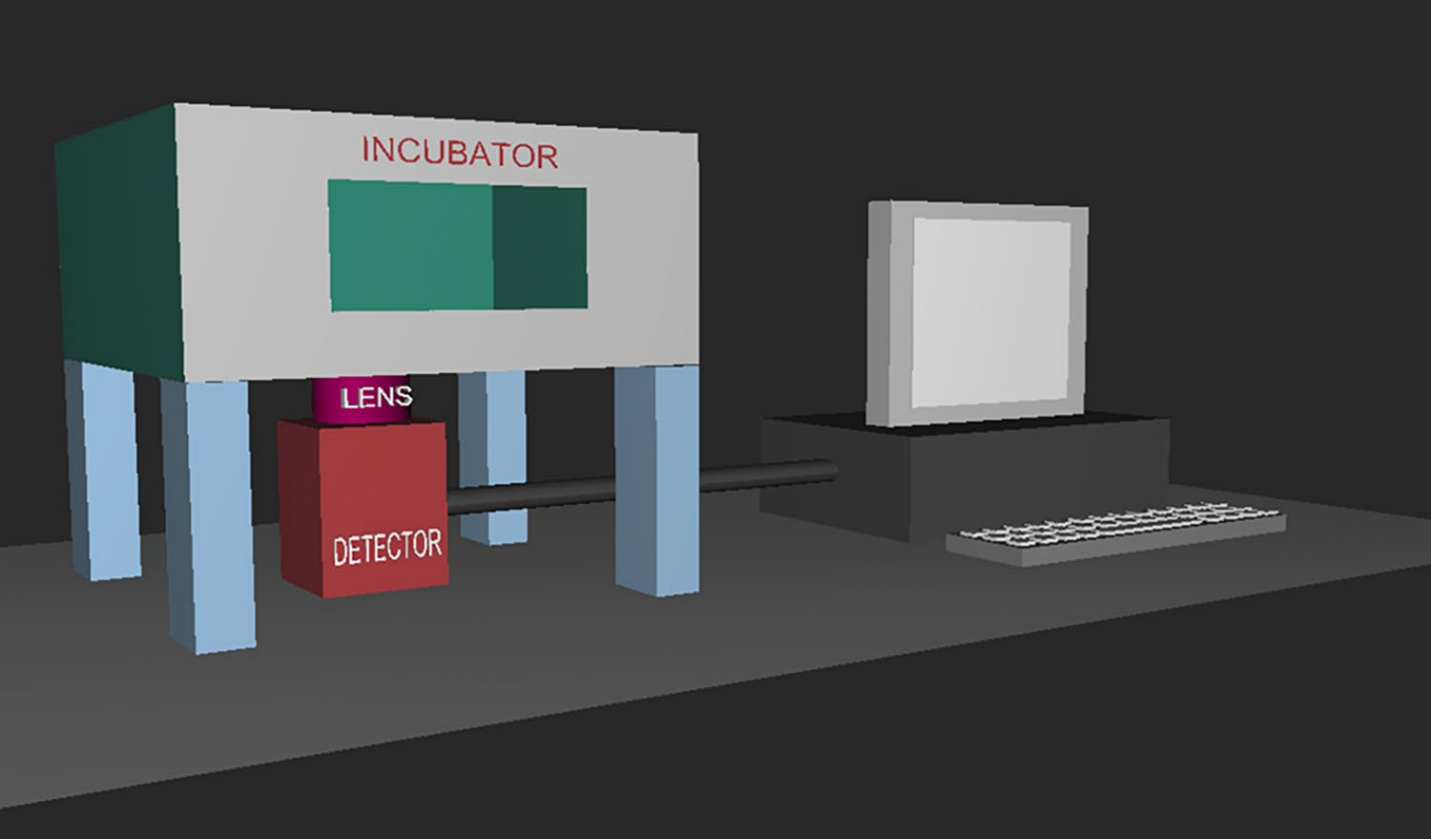
Arrangement of the CMOS camera, microscope incubator and computer instrument complex.

**Figure 10:**
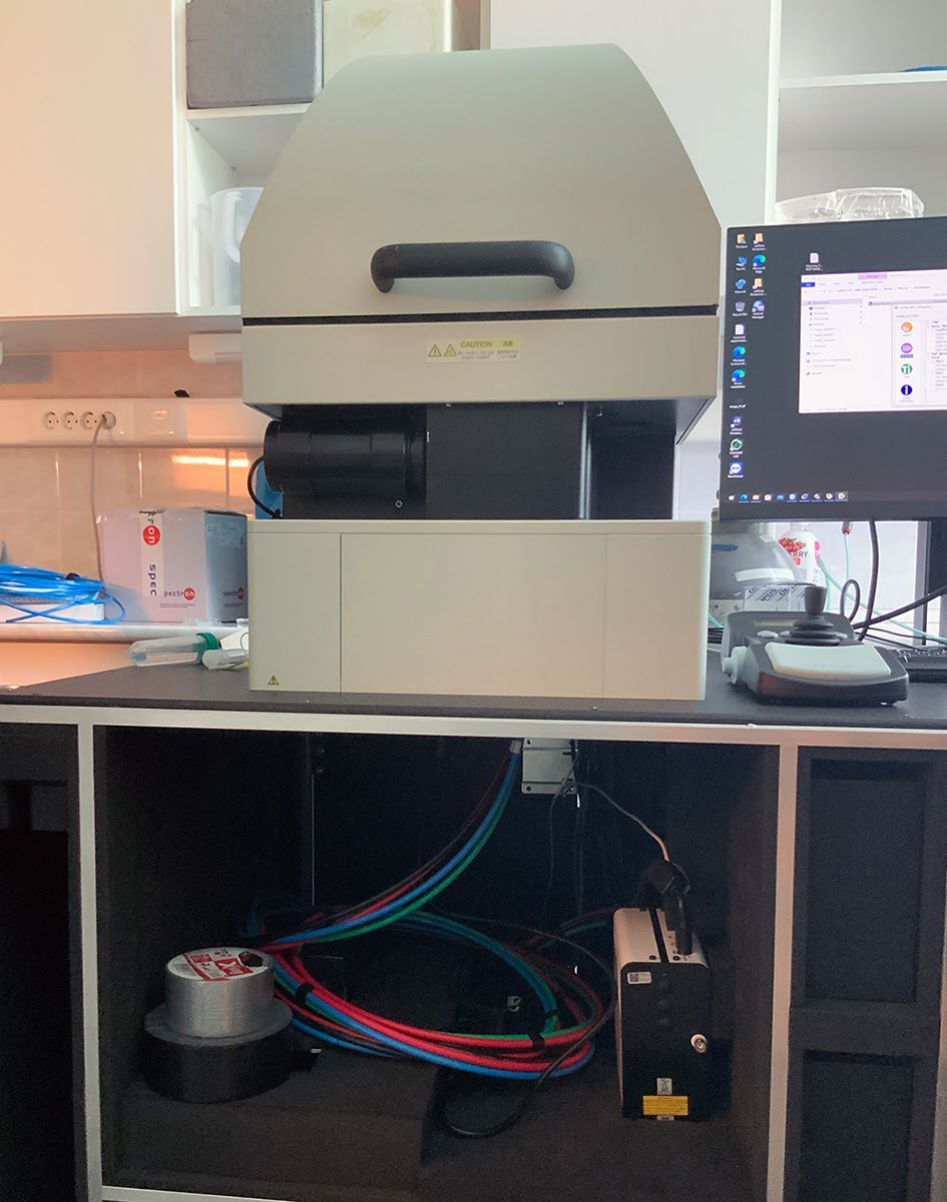
Photograph of the CMOS camera, microscope incubator and computer instrument complex.

UPE detection was performed using the ORCA-Quest CMOS camera (Hamamatsu Photonics), which is an extremely sensitive device that can detect single photons, and was used to observe time-dependent photon emission intensity. The system is equipped with a quantitative CMOS image sensor providing a maximum spectral response from 300 to 1000 nm, cooled to − 20 °C. The effective number of pixels was 4096 (H) × 2304 (V), the pixel size was 4.6 × 4.6 µm and the quantum efficiency was 90% at 475 nm and 33% at 900 nm. The camera was used with a microscope incubator (Olympus), which ensured the ideal conditions for embryo cultivation at 37 °C while allowing the detection of photons emitted by the embryos by excluding visible light (i.e. creating completely dark conditions; Figs. 9 and 10). We studied the photon emission of freshly conceived and frozen/thawed embryos.

**Figure 11:**
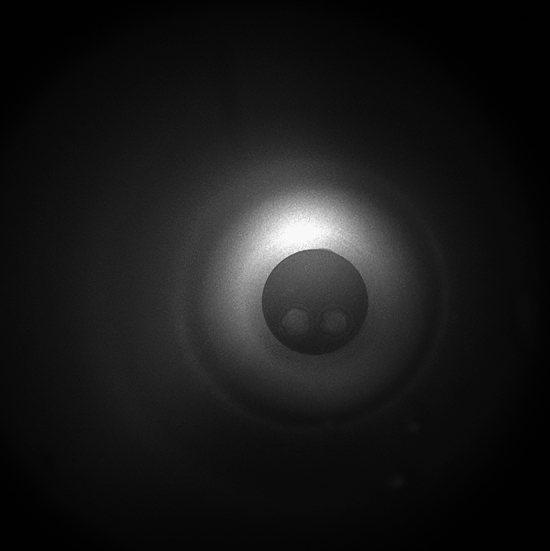
First frame of 50 h measurement.

**Figure 12:**
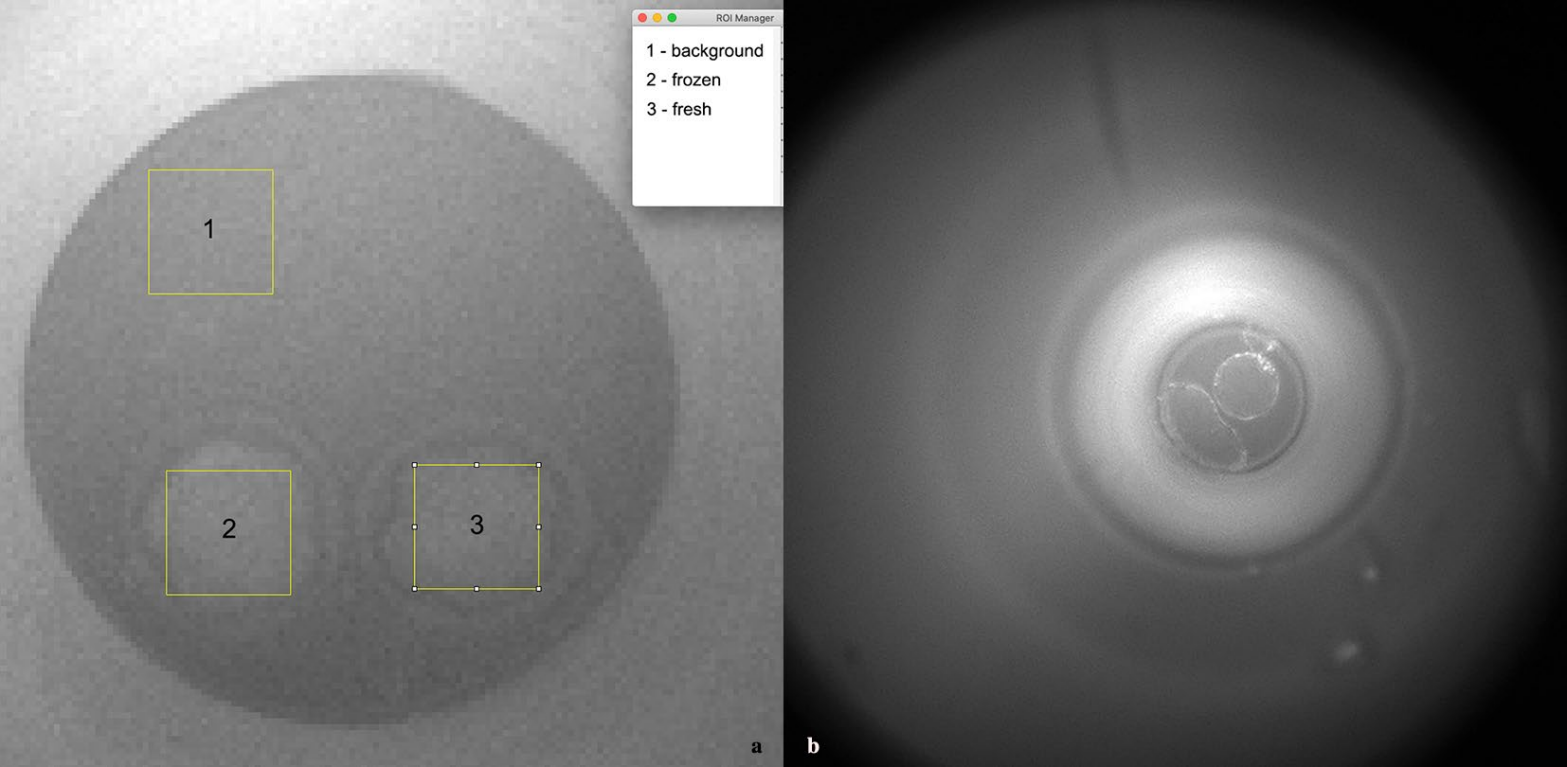
(**a**) Designation of sampling areas on the illuminated image taken before measurement: (1) background, (2) frozen embryo and (3) fresh embryo. (**b**) Last frame of 50 h measurement, embryos at blastula and hatching stages.

**Figure 13:**
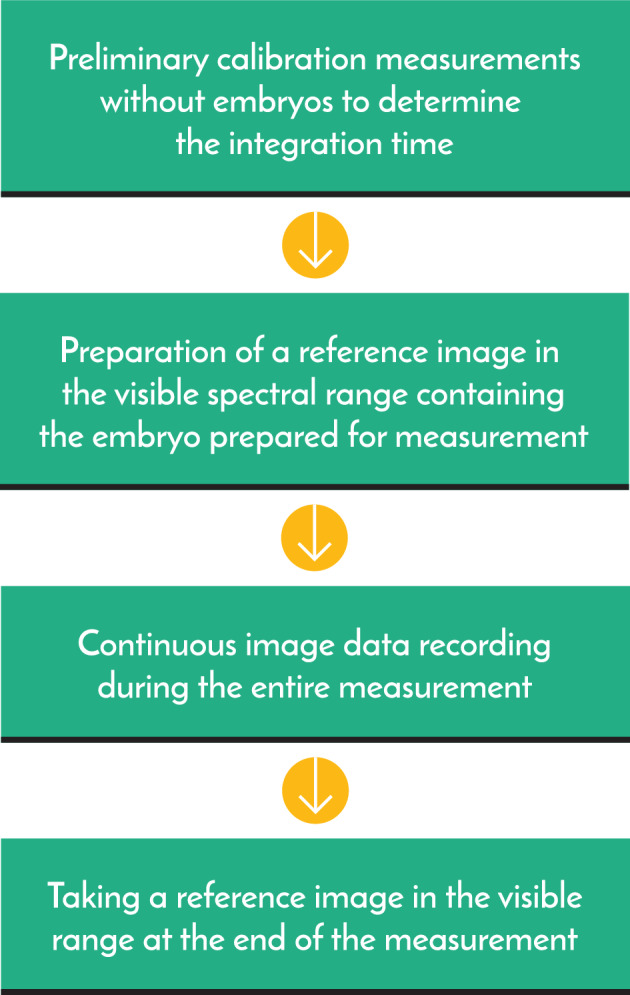
The preparation before the measurement and a schematic presentation of the most important steps of the measurement.

Data were collected continuously during the measurement period. Before and immediately after measurement, the following reference recordings were made under visible conditions to aid setting and processing (Figs. 11, 12, 13): the completely empty incubator and the measurement space containing, respectively, an empty sample holder, a sample holder and incubation medium, and an embryo in incubation medium. For the basic measurement of the completely empty incubator, the camera settings were changed so that the intensity of each pixel equalled zero: we disabled automatic amplification, started cooling based on the Peltier effect, and stabilised when the sensor had reached an operating temperature of − 20 °C. Illustration of the results of the reference measurement with the sample holder and sample holder with incubation medium placed in the measurement space based on EW-SFD values (Eq. 11). At the next step of measurements of the non-working diode (blue curve) placed in the measurement space, as well as the continuously working red diode (red curve), the intensity values from the live embryo, all of which were corrected with the dark data of the sensor operating at − 20 °C. In the second period, we deactivated all filters in the measurement software to maximise the data.

The establishment of the integration time is an important component of accurate measurement. If the image-sensing device behaved ideally, the shortest factory-established integration time would be appropriate. In practice, the determination of the device setting based on real conditions (the characteristics of the camera, measurement space and object) is advisable. The following factors were considered when determining the final integration time: the camera amplification, photons reflected within the measuring space, photons generated in the measurement space and not emitted by the embryo, photons entering the measurement space from the external environment, strength and temporal inhomogeneity of photons emitted by the embryo, and data backup parameters employed during long-term measurement. The deactivation of the camera's amplification eliminated non-zero intensity values caused by amplification during measurement of the empty space, significantly reducing disturbance from photons not emitted by the embryo. Regarding the backup parameters, the system had a 2-GB file size limit for control and data saving, which allowed for up to 50 h of data recording, even with the application of a minute-by-minute integration time (3000 images). Thus, photons emitted by the embryo could be detected with minimal noise, representing physiologically justified sampling. Figure 13 summarizes the most important steps of the measurement and its preparation.

### Image processing

**Figure 14:**
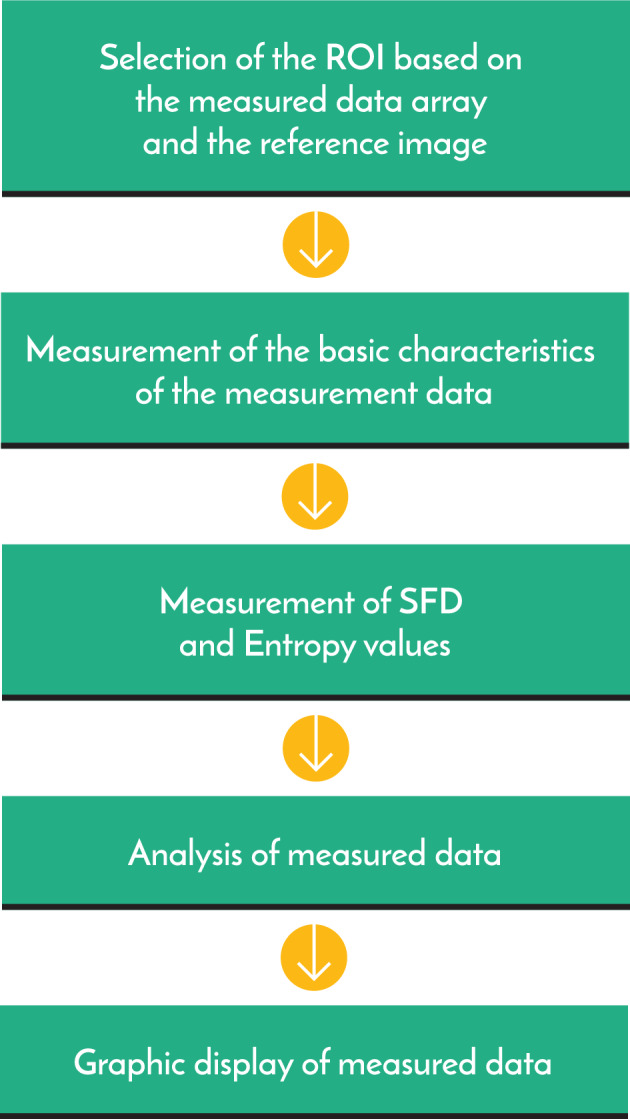
Presentation of the most important elements of data processing.

The digital image data generated during measurement were saved batch lossless format (.btf) with a depth of 16 bits/pixel data and integration time of 1 min. During the data processing, based on the reference images taken in the visible range, areas of the same size (ROI—Region of Interest) were selected for accurate measurement of the objects. After selecting areas of the same size, we performed a pixel-by-pixel correction with the background data (Fig. 12, region 1), after them entropy-, SFD- and EW-SFD-based measurements were performed. Figure 14 illustrates the schematic presentation of data processing.

### Institutional review board statement

All experimental protocols were approved by the Regional and Local Research Ethics Committee of the University of Pécs, Pécs, Hungary (PTE KK 7072-2018). The study is reported in accordance with ARRIVE guidelines. All methods were carried out in accordance with relevant guidlines and regulations.

## Data Availability

The original data collected during the study are available from the corresponding author upon request.
